# Myelin Imaging of the Spinal Cord in Animal Models and Patients with Multiple Sclerosis Using [^11^C]MeDAS PET: A Translational Study

**DOI:** 10.2967/jnumed.123.266896

**Published:** 2025-01

**Authors:** Chris W.J. van der Weijden, Ahmed K.M.A. Ahmed, Anouk van der Hoorn, Junqing Zhu, Chunying Wu, Yanming Wang, Gilles N. Stormezand, Rudi A.J.O. Dierckx, Jan F. Meilof, Erik F.J. de Vries

**Affiliations:** 1 Nuclear Medicine and Molecular Imaging, University Medical Center Groningen, University of Groningen, Groningen, The Netherlands;; 2 Department of Radiology, University Medical Center Groningen, University of Groningen, Groningen, The Netherlands;; 3 Department of Molecular Neuroscience, Osaka University, Suita, Japan;; 4 WPI Immunology Frontier Research Center, Osaka University, Suita, Japan;; 5 Department of Radiology, Case Western Reserve University, Cleveland, Ohio;; 6 Department of Neurology, Martini Ziekenhuis, Groningen, The Netherlands;; 7 Department of Biomedical Sciences, University Medical Center Groningen, University of Groningen, Groningen, The Netherlands; and; 8 Multiple Sclerosis Center Noord Nederland, Groningen, The Netherlands

**Keywords:** [^11^C]MeDAS, spinal cord, multiple sclerosis, myelin, PET

## Abstract

Multiple sclerosis (MS) is a neurodegenerative disease characterized by demyelinated lesions in the brain and spinal cord. A few clinical studies using PET to image myelin in the brain have been performed, but none investigated the spinal cord. Because clinically relevant motor symptoms are primarily due to spinal cord damage, this translational study evaluated [^11^C]*N*-methyl-4,4′-diaminostilbene (MeDAS) as a PET tracer for myelin imaging in the rat and human spinal cord. **Methods:** [^11^C]MeDAS PET of the spinal cord was conducted in experimental autoimmune encephalomyelitis, lysophosphatidylcholine, and spinal cord injury animal models of focal demyelination. Then, 6 healthy controls and 11 MS patients were subjected to MRI and [^11^C]MeDAS PET of the spinal cord between C5 and T6 vertebrae. Regions of interest covering 100%, 60%, and 40% of the diameter of the spinal canal were drawn, and tracer uptake was normalized to the activity in the blood pool, muscle, or injected dose per unit of body weight (SUV). **Results:** [^11^C]MeDAS uptake was significantly reduced in spinal cord lesions in all animal models. In humans, tracer uptake was significantly higher in the cervical than the thoracic spinal cord, which corresponds well with the known physiologic rostral–caudal gradient in myelin density. MS patients had significantly lower [^11^C]MeDAS uptake in the upper spinal cord (C5–T3) than did controls. The [^11^C]MeDAS PET signal was inversely correlated with the presence of MS lesions in specific sections of the spinal cord. The best differentiation among regions with different myelin density was obtained when the smallest region of interest was used and spinal cord uptake was expressed as SUV. **Conclusion:** [^11^C]MeDAS PET shows the potential to quantify myelin density in the spinal cord. It enables detection of physiologic differences in myelin density between spinal cord segments and between MS patients and healthy controls, which warrants further evaluation of this technique.

## Visual Abstract

**Figure:**
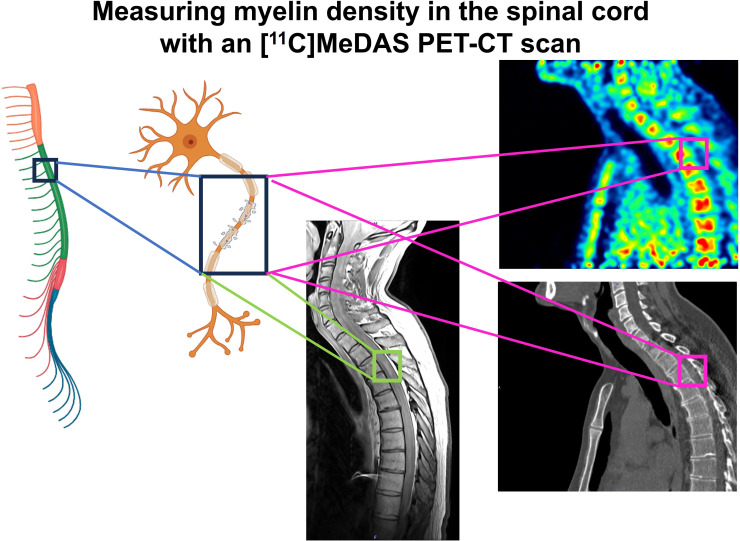

Multiple sclerosis (MS) is the most common neurodegenerative disease among young adults. MS has both inflammatory and demyelinating aspects (1). Inflammation results in demyelinated and degenerated lesions, called plaques (2). Myelin, which is wrapped around neuronal axons, has neuroprotective, neurotransmission-enhancing, and axonal metabolic support functions (3*,*^4^). Disturbances in myelin integrity decrease the efficacy of neuronal functions and make neurons susceptible to degeneration. Restoration of the myelin sheath could restore neuronal functions and promote neuronal survival. Therefore, research into the development of remyelination therapies is relevant. However, an accurate biomarker to evaluate the efficacy of such remyelination strategies is missing.

In vivo characterization of myelin damage with the help of imaging techniques would be an attractive tool for the evaluation of remyelination therapies. Several MRI techniques for myelin imaging have been developed. The accuracy of these methods for imaging myelin has recently been assessed (5*,*^6^). The main conclusion was that current myelin MRI methods do not suffice for accurate quantitative myelin imaging. Several PET tracers have been developed with the aim to depict myelin content in vivo (7–10). In particular, several amyloid PET tracers have been repurposed for this task, including ^11^C-labeled Pittsburgh compound B and [^18^F]florbetapir (7–10). These tracers have shown promising results in preclinical studies, and some have recently been used in clinical studies (11–14). The quick advancement of the repurposed amyloid tracers for myelin imaging in clinical studies mainly occurs because amyloid tracers (in particular, ^11^C-labeled Pittsburgh compound B and [^18^F]florbetapir) have already been routinely used for diagnosis of Alzheimer disease and thus are readily available.

Our research has focused on tracers with a central stilbene structure that were specifically developed for myelin imaging. A promising candidate among these tracers is [^11^C]*N*-methyl-4,4′-diaminostilbene (MeDAS). In preclinical studies, [^11^C]MeDAS PET showed higher accuracy than ^11^C-labeled Pittsburgh compound B PET for imaging myelin (7). Furthermore, competitive binding assays of [^3^H]MeDAS using isolated myelin fractions showed specific binding, whereas no binding of [^3^H]MeDAS was observed in isolated fractions that were devoid of myelin (15*,*^16^). In addition, when the fluorescence characteristics of MeDAS in rats were exploited, MeDAS staining of myelin with fluorescence microscopy was virtually identical to that of immunohistochemical staining of myelin basic protein (10*,*^15^). Distinct [^11^C]MeDAS binding to myelin in the corpus callosum of mice could be blocked with the myelin-binding compound 1,4-bis(diphenylamino)benzene (at 5 mg/kg), suggesting that [^11^C]MeDAS binds to myelin with high selectivity and specificity (10). Recently, we performed a first-in-human study with [^11^C]MeDAS PET, showing that the method is suitable for imaging of myelin density in the brain (17*,*^18^).

However, no studies on PET imaging of myelin in the human spinal cord have been performed so far. In particular, in the later progressive stages of MS, demyelination and degeneration of the spinal cord are the main drivers of disability. Therefore, this study aimed to assess the feasibility of [^11^C]MeDAS PET for imaging of myelin in the spinal cord in various animal models and subsequently in human subjects.

## MATERIALS AND METHODS

### Animal Experiments

A detailed description of the experimental autoimmune encephalomyelitis, lysophosphatidylcholine, and spinal cord injury rat models for demyelination is provided in the supplemental materials (supplemental materials are available at http://jnm.snmjournals.org). Under anesthesia (4% isoflurane), approximately 37 MBq of [^11^C]MeDAS (44,400 GBq/mmol) were injected via a tail vein, and immediately, a 60-min dynamic PET acquisition (Siemens Inveon PET/CT with a field of view of 12.7 cm) was started. Subsequently, a 2-bed-position CT scan was performed that covered the spinal cord from T2 to L3. A 2-dimensional ordered-subset expectation maximization algorithm was applied for image reconstruction (5 frames of 60 s and 11 frames of 300 s), using CT as attenuation correction. The PET images were registered to the CT images, which enabled an accurate definition of the region of interest (ROI) per individual vertebra (T2–L3). Radioactivity concentrations in each ROI were determined 40–60 min after tracer injection and normalized to average T2–L3 spine uptake. No a priori inclusion or exclusion criteria were defined for the analysis of animal data. No animals were excluded for the quantitative analysis of [^11^C]MeDAS PET.

### Clinical Studies

#### Subjects

Six healthy controls (HCs) and 11 MS patients diagnosed according to the revised McDonald criteria were included in this prospective study. The MS patients were enrolled at either the Martini Hospital or the University Medical Center Groningen. HCs were recruited via advertisements in social media. Because this is a pilot study, no group size calculation was performed. The inclusion criteria for patients were at least 18 y of age and a diagnosis of progressive MS for at least 5 y. HCs were age-matched. Exclusion criteria for all participants included pregnancy, breastfeeding, claustrophobia, cerebrovascular disease, or a clinical history of diminished renal or liver function. The study was approved by the institutional review board of the University Medical Center Groningen (METc 2018/450; Netherlands Trial register, Trial NL7262), and all subjects signed an informed-consent form.

#### Image Acquisition

Structural, sagittal, 2-dimensional, and T2-weighted (T_2_w) MRI scans (repetition time, 3,500 ms; echo time, 85 ms; 17 slices, 3-mm slice thickness; flip angle, 160°; voxel size, 0.4 × 0.4 × 3.0 mm) and sagittal, 2-dimensional, and proton density–weighted MRI scans (repetition time, 2,500 ms; echo time, 7.7 ms; 15 slices, 3-mm slice thickness; flip angle, 160°; voxel size, 0.4 × 0.4 × 3.0 mm) of the spinal cord (vertebrae C5–T6) were acquired on a 3.0-T Prisma scanner (Siemens Healthineers). Static PET scans of the spinal cord were acquired on a Siemens Biograph Vision PET/CT scanner, with a field of view of 25.6 cm, 60 min after injection of [^11^C]MeDAS (HCs, 203 ± 46 MBq, 4.2 ± 0.9 µg; MS patients, 209 ± 35 MBq, 2.7 ± 1.3 µg). Scan procedures started with a low-dose CT scan and were followed by a 10-min static [^11^C]MeDAS PET scan, covering vertebrae C5–T6. PET images were corrected for attenuation, randoms, dead time, scatter, and decay. For 1 subject, the aorta and heart were not within the field of view of the PET/CT scan, and for another subject, C5 and C6 were not in the field of view of the PET/CT scan.

#### Image Analysis

Low-dose CT was used instead of MRI to segment the spinal cord because of differences in the positioning of the subjects between scanners. The 3-dimensional ROIs for the spinal cord, divided into sections corresponding to the vertebrae, were manually drawn in PMOD version 4.1 (PMOD Technologies Ltd.). ROIs covering 100%, 60%, and 40% of the diameter of the spinal canal were drawn to investigate potential spill-in effects (Supplemental Fig. 1). ROI volumes for each vertebra section are provided in Supplemental Table 1. The spinal cord ROIs corresponding to C5–C7, T1–T3, and T4–T6 were combined into 3 new ROIs to assess whether [^11^C]MeDAS PET could detect the physiologic decrease in myelin density from the cervical toward the caudal spinal cord (19). Spinal cord uptake was corrected for net injected dose and body weight and was expressed as SUV. In addition, ROIs were drawn for the blood pool of the heart, aorta, and neck muscle, which were used to calculate spinal cord ratios (SCRs).

The T_2_w and proton density–weighted MR images were used for lesion detection by an experienced neuroradiologist. The lesions are characterized as hyperintensities on T_2_w and proton density–weighted MRI. Subsequently, the correspondence between the segments with a significantly decreased myelin PET signal in MS patients (compared with HCs) and the presence of lesions detected with MRI (1 or plural) in these segments was assessed.

#### Statistics

Statistical analyses were performed in SPSS version 23 (IBM). For animal studies, paired *t* tests were performed to evaluate differences in tracer uptake between baseline and after lesion induction. A *P* value of less than 0.05 was accepted as significant. For human studies, [^11^C]MeDAS uptake in the spinal cord regions was assessed for normality and analyzed with ANOVA and a post hoc least significant difference test to determine whether cervical PET tracer uptake is higher than thoracic PET tracer uptake. A group-based comparison using a 2-tailed *t* test was performed to assess whether MS patients had significantly different tracer uptake per vertebra section than HCs. A Cohen *D* value was used to express the effect sizes of these differences. The 95% CI of [^11^C]MeDAS uptake in HCs was calculated to assess whether individual MS patients had significantly different PET tracer uptake per vertebra section compared with HCs (i.e., outside the 95% CI of HCs). The correspondence between the regions with significantly different PET tracer uptake in MS patients and the presence of spinal lesions on MRI at the same vertebra level was assessed. This information was used to calculate the negative predictive value (NPV), positive predictive value (PPV), sensitivity, and specificity of [^11^C]MeDAS PET. Because this is an explorative study, investigating multiple parameters for measuring [^11^C]MeDAS uptake in the spinal cord, no corrections for multiple comparisons have been performed.

## RESULTS

### Preclinical Studies

**FIGURE 1.**
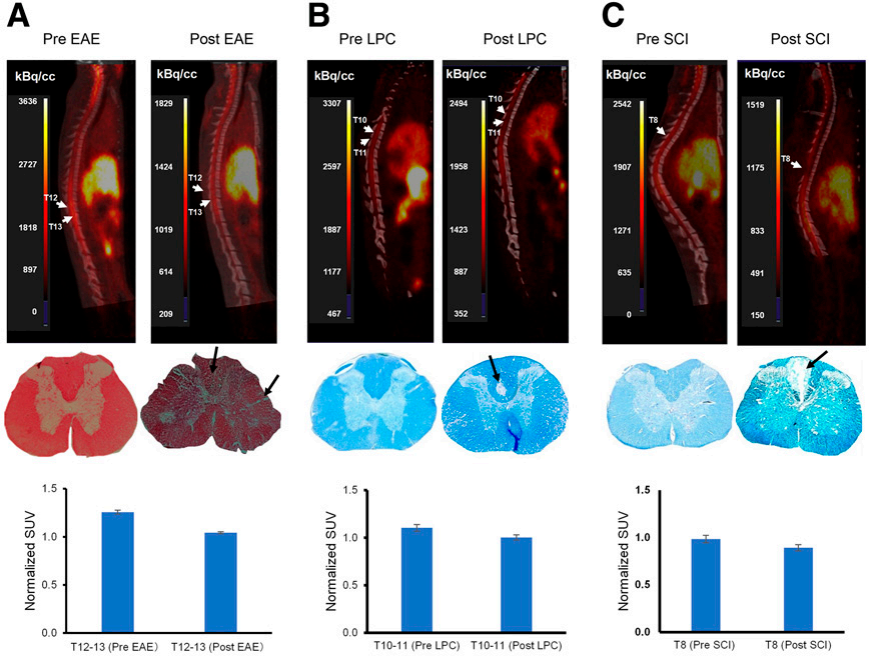
(Top) Representative [^11^C]MeDAS PET images of spinal cord in experimental autoimmune encephalomyelitis (EAE; A), lysophosphatidylcholine (LPC; B), and spinal cord injury (SCI; C) rat models of focal demyelination, displayed both at baseline and after lesion induction. (Middle) Corresponding pathologic characterization of typical lesion region in same PET cohort of each animal model, with specific area marked by arrow for clarity. (Bottom) Tracer uptake in lesion region, expressed as normalized SUV ratio, before and after lesion formation.

[^11^C]MeDAS PET for myelin in the spinal cord was performed in the experimental autoimmune encephalomyelitis, lysophosphatidylcholine, and contusion spinal cord injury rat models of focal demyelination. As shown in Figure 1, the spinal cord could be visualized with high resolution with [^11^C]MeDAS PET. In all animal models, [^11^C]MeDAS uptake in the affected spinal cord region was significantly lower after lesion induction than at baseline (Fig. 1; Supplemental Table 2). In the experimental autoimmune encephalomyelitis rat model, [^11^C]MeDAS uptake in T12–T13 was 17% lower than at baseline (SUV ratio for the T2–L3 spine, 1.25 ± 0.02 vs. 1.04 ± 0.01; *n* = 6; *P* = 0.011), even though the small lesions were not visible with PET. Similar reductions in [^11^C]MeDAS uptake in the spinal cord lesions were observed in both the lysophosphatidylcholine model (SUV ratio for the T2–L3 spine, 1.10 ± 0.09 vs. 1.00 ± 0.03; *n* = 5; *P* = 0.042) and the spinal cord injury model (SUV ratio for the T2–L3 spine, 0.98 ± 0.05 vs. 0.89 ± 0.02; *n* = 5; *P* = 0.018).

### Clinical Studies: Demographics

One MS patient was excluded from the analysis because only C1–T1 was imaged; another MS patient who was imaged from C7 to T9 was also excluded. No significant differences in age and sex between HCs and MS patients were observed (Supplemental Table 3). MRI detected MS lesions in patients at all spinal cord levels defined by the vertebra (Supplemental Fig. 2). The least frequently affected spinal levels were T1, T3, T4, and T5, whereas T6 was the most frequently affected. In this population, 45% of the lesions were located in the cervical spinal cord (C5–C7) and 55% of the lesions were located in the thoracic spinal cord (T1–T6).

### Myelin Gradient in Spinal Cord of HCs

**FIGURE 2.**
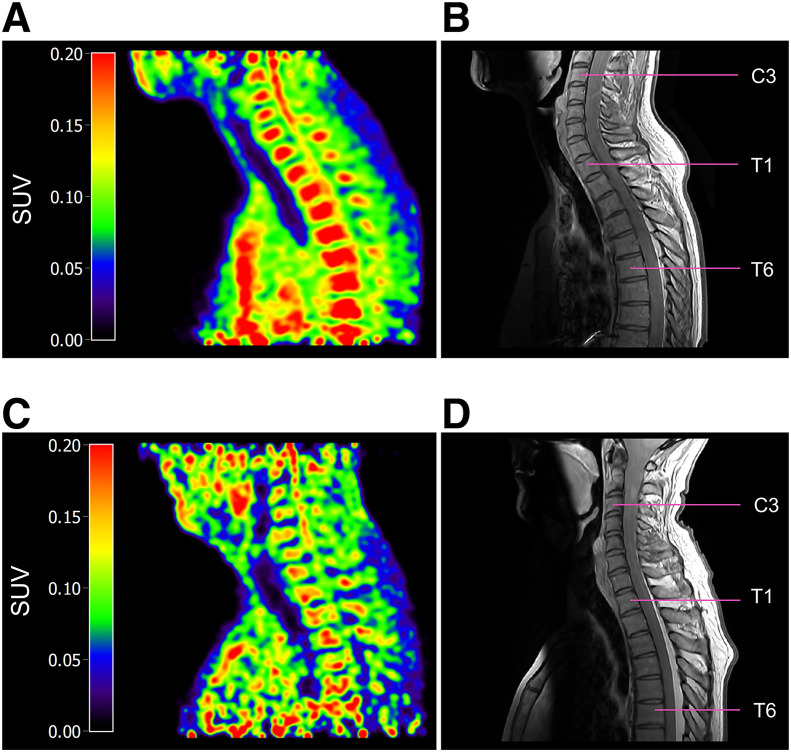
Sagittal [^11^C]MeDAS PET of cervical spinal cord of HC and MS patient (5 iterations, 5-mm gaussian filter). (A) [^11^C]MeDAS PET scan of spinal cord from C3 to T8 of HC. (B) Proton density–weighted MRI of spinal cord from C3 to T8 of same HC. (C) [^11^C]MeDAS PET scan of spinal cord from C1 to T6 of MS patient. (D) Proton density–weighted MRI of spinal cord from C1 to T6 of same MS patient.

[^11^C]MeDAS PET images of the spinal cord of HCs show the highest tracer uptake in the cervical spinal cord (Fig. 2), with tracer uptake decreasing in more caudal segments. This corresponds well with the known physiologic rostral–caudal myelin gradient (19). High uptake of the tracer is visible within the vertebrae, which was not observed in animals. No adverse advents because of the [^11^C]MeDAS PET scan were reported.

PET data of HCs were used to determine the optimal parameter to reproduce the physiologic myelin gradient in the spinal cord, using SUV and SUV ratios (SCR heart, SCR aorta, and SCR muscle) from ROIs covering 100%, 60%, and 40% of the diameter of the spinal canal. For the 40% and 60% ROIs, a significant difference in tracer uptake between the cervical and the thoracic spinal cord was observed, but not between the upper and the lower thoracic spinal cord, irrespective of whether tracer uptake was expressed as SUV, SCR heart, SCR aorta, or SCR muscle (Table 1; Supplemental Table 4). For the 100% ROI, the SCR muscle was significantly different only between the cervical and the thoracic spinal cord, not between the upper and the lower thoracic spinal cord. The best discrimination between tracer uptake in the cervical and that in the thoracic spinal cord (i.e., largest *F* values and smallest *P* values) was obtained for the smallest spinal cord ROI (40%). These results suggest that considerable partial-volume effects occurred when the 100% ROI was used; therefore, we eliminated the results from the 100% ROI from further analyses.

**TABLE 1.** [^11^C]MeDAS Uptake in Sections of Spinal Cord of HCs, Corresponding to C5–C7, T1–T3, and T4–T6

Parameter	ROI size (%)	C5–C7	T1–T3	T4–T6	*F* ANOVA	*P*
SUV	100	1.02 ± 0.25	0.80 ± 0.13	0.78 ± 0.13	1.88	0.195
	60	1.23 ± 0.31	0.85 ± 0.17	0.81 ± 0.14	4.72	0.031*
	40	1.41 ± 0.29	0.92 ± 0.18	0.83 ± 0.15	9.02	0.004*
SCR aorta	100	1.12 ± 0.13	0.89 ± 0.17	0.88 ± 0.19	3.60	0.059
	60	1.35 ± 0.16	0.95 ± 0.18	0.91 ± 0.20	11.18	0.002*
	40	1.55 ± 0.15	1.02 ± 0.18	0.93 ± 0.20	24.46	>0.001*
SCR heart	100	1.15 ± 0.12	0.92 ± 0.18	0.91 ± 0.21	2.45	0.142
	60	1.39 ± 0.15	0.98 ± 0.19	0.94 ± 0.22	7.40	0.013*
	40	1.60 ± 0.15	1.05 ± 0.18	0.96 ± 0.21	15.03	0.001*
SCR muscle	100	1.42 ± 0.14	1.13 ± 0.16	1.11 ± 0.19	5.26	0.023*
	60	1.72 ± 0.17	1.20 ± 0.18	1.15 ± 0.21	16.33	>0.001*
	40	1.97 ± 0.12	1.29 ± 0.19	1.18 ± 0.20	38.03	>0.001*

* Statistically significant.

Data are presented as mean ± SD. Between group degrees of freedom (*F*) are 2 for all analysis in Table 1.

### [^11^C]MeDAS PET in MS Patients

In MS patients, both 60% and 40% SUVs were significantly different between the cervical spinal cord (C5–C7) and the upper (T1–T3) or lower (T4–T6) part of the thoracic spinal cord (Supplemental Tables 5 and 6). The same trend was observed for SUV ratios, with the exception of SCR heart (60%), which did not show a significant difference between spinal cord segments.

When tracer uptake in the spinal cord was compared between HCs and MS patients (Table 2; Supplemental Table 7), significant differences were observed in all segments when uptake was expresses as SUV using a 40% ROI. For SUVs from a 60% ROI, significant differences between HCs and MS patients were found only in segments C5–C7 and T1–T3. No significant differences were observed when SUV ratios were used (Supplemental Table 8), which might be due to the extra variation introduced by the reference tissues.

**TABLE 2.** Statistical Analysis of Differences in [^11^C]MeDAS Uptake in Spinal Cord, Expressed as SUV, Between HCs and MS Patients

Spinal cord section	ROI size (%)	HCs	MS patients	Cohen *D* effect size	*T*	*P*
C5–C7	60	1.23 ± 0.31	0.78 ± 0.13	1.39	3.32	0.006*
	40	1.41 ± 0.29	0.86 ± 0.15	1.53	4.09	0.001*
T1–T3	60	0.85 ± 0.17	0.56 ± 0.17	1.17	2.48	0.029*
	40	0.92 ± 0.18	0.58 ± 0.18	1.28	2.87	0.014*
T4–T6	60	0.81 ± 0.14	0.54 ± 0.20	1.05	2.12	0.055
	40	0.83 ± 0.15	0.55 ± 0.21	1.07	2.18	0.050

* Statistically significant.

Data are presented as mean ± SD. *T* is derived from *T*-test.

### Assessment of Myelin Loss

[^11^C]MeDAS uptake in the spinal cord section corresponding to a specific vertebra was considered significantly different between a MS patient and the HC group if tracer uptake of that MS patient was outside the 95% CI of the HC group. The sections with a significant difference in tracer uptake between the individual MS patient and the HC group were correlated with the presence of a lesion in that segment on the MRI scan of that MS patient. On the basis of the correspondence between PET and MRI, the NPV, PPV, sensitivity, and specificity of [^11^C]MeDAS PET for detection of lesions were determined. All PET measures yielded moderate PPV, good NPV, poor sensitivity, and good specificity (Table 3).

**TABLE 3.** Correspondence of Abnormal [^11^C]MeDAS Spinal Cord Uptake with Presence of Lesion According to MRI

Parameter	ROI size (%)	PPV (%)	NPV (%)	Sensitivity (%)	Specificity (%)
SUV	60	50	80	20	94
	40	38	80	25	88
SCR aorta	60	56	85	45	90
	40	57	84	40	91
SCR heart	60	43	85	50	81
	40	36	82	40	79
SCR muscle	60	50	81	25	93
	40	56	81	25	94

## DISCUSSION

Quantitative characterization of myelin density could aid in the development of myelin repair treatments and monitoring of disease progression in patients with MS. Because MS pathology located in the spinal cord is most relevant for development of clinical impairments, the aim of this translational study was to assess the feasibility of imaging myelin density in the spinal cord with [^11^C]MeDAS PET in animal models and humans.

Our preclinical studies demonstrated that [^11^C]MeDAS PET can detect demyelinated lesions in the spinal cord across various rat models using different methods to induce demyelination. In all models, reduced [^11^C]MeDAS uptake was consistently observed in affected spinal cord regions. High-contrast visualization of the spinal cord was achieved despite its small diameter in rats. In the experimental autoimmune encephalomyelitis rat model, in which small lesions were not visible, a significant reduction in [^11^C]MeDAS uptake was detected in the T12–T13 region. This suggests that [^11^C]MeDAS PET may be sensitive enough to detect subtle changes in myelin content, making it valuable for early detection and monitoring of demyelination. Similar reductions in [^11^C]MeDAS uptake were observed in lysophosphatidylcholine-induced demyelination and spinal cord injury models, indicating that the findings are independent of demyelination mechanisms. Our results correspond with other studies assessing [^11^C]MeDAS PET in an animal spinal cord (7*,*^12^). These promising preclinical results prompted us to evaluate the feasibility of [^11^C]MeDAS PET assessment of the myelin density of the human spinal cord.

In our clinical studies, various normalization strategies and ROI sizes (i.e., percentage of the diameter of the spinal canal) were evaluated to determine the optimal parameters for quantifying [^11^C]MeDAS uptake. Although SUV is commonly used, it is susceptible to confounding factors. To compensate for this, SUV ratios with the aorta, blood pool of the heart, or neck muscle were evaluated. These normalization methods differ in their susceptibility to partial-volume effects, spillover effects, motion, or flow artifacts, which might all influence the accuracy of [^11^C]MeDAS PET quantification in the spinal cord. Irrespective of the outcome parameter used, [^11^C]MeDAS uptake showed a gradient in the spinal cord that aligned with the known myelin gradient (19). SCR muscle had the highest discriminative power between spinal cord segments in HCs, whereas SUV best distinguished differences in myelin density between spinal cord segments in MS patients and differentiated MS patients from HCs. Therefore, SUV seems to be the preferred parameter for quantifying myelin density in static [^11^C]MeDAS PET images of the spinal cord, over the ratio methods.

Differences in [^11^C]MeDAS uptake in the spinal cord between MS patients and HCs are even present in spinal cord segments without overt MS lesions on MRI (Supplemental Table 9), suggesting early demyelination may occur before visible MRI changes. This finding is similar to that for the normal-appearing white matter in the brain of MS patients, which was suggested to have lower myelin density than normal-appearing white matter in HCs. However, on an individual level, this effect was less clear, as indicated by the high specificity (Table 3), likely due to the small number of HC participants and the resulting large 95% CIs, which hampered detection of abnormal tracer uptake in MS patients.

Delineating the spinal cord on [^11^C]MeDAS PET or CT images is challenging, although vertebrae are well defined on low-dose CT images. ROIs of 100%, 60%, and 40% spinal canal diameter were tested as spinal cord proxies. Larger ROIs showed no significant differences in [^11^C]MeDAS uptake between the cervical and the thoracic regions, which contradicts known differences in myelin density. [^11^C]MeDAS uptake in the 40% ROI of the spinal canal diameter best reflected the myelin density gradient in the spinal cord of HCs (highest *F* values). Even with MS lesions, smaller ROIs enabled detection of the myelin density gradient in MS patients. Large ROIs apparently suffered substantially from spillover effects and therefore were excluded from further analysis. Thus, only 60% and 40% ROIs were further evaluated, acknowledging the noise sensitivity of these smaller ROIs. Partial-volume correction might enhance accuracy by addressing spill-out effects and underestimation of spinal cord radioactivity concentration.

A 40% or 60% SUV showed high specificity (88%–94%) and NPV (80%) in detecting abnormal myelin density with [^11^C]MeDAS PET, indicating a strong correspondence between PET and MRI, especially when lesions are absent. However, sensitivity (20%–25%) and PPV (38%–50%) were relativity low for detecting myelin abnormalities. The moderate PPV of [^11^C]MeDAS PET suggests that [^11^C]MeDAS PET detects more abnormalities than the lesions detected with MRI. This may be explained by spinal cord atrophy, which is even more common than brain atrophy in MS patients (20). Another explanation might be the presence of diffuse pathology in the spinal cord beyond MS lesions, as can be seen using proton density–weighted MRI, especially in patients with primary progressive MS (21). This diffuse pathology is not rated as an individual lesion on T_2_w MRI but likely correlates with lower myelin densities, which would explain the [^11^C]MeDAS PET findings.

The low sensitivity indicates that many MRI lesions were not detected by PET. Preclinical studies indicated that [^11^C]MeDAS PET is unaffected by neuroinflammation (16). Early-stage lesions with inflammation but minimal demyelination might be visible on MRI but not on PET. Furthermore, fully remyelinated lesions on T_2_w MRI likely show normal [^11^C]MeDAS uptake. Differences in lesion count between PET and MRI could also result from the small lesion size, which is below the spatial resolution of the PET camera. Another explanation could be our analysis method. In the PET analysis, we examined not individual lesions but rather decreases in tracer uptake in spinal segments defined by vertebrae, because lesions were not visible on the PET scan due to their small size and because coregistration of PET with MRI was not possible due to the different orientations of the spinal cord between the 2 imaging techniques. Future studies could benefit from using hybrid PET/MRI cameras, which would allow simultaneous PET and MRI acquisition and mitigate alignment issues between the 2 modalities. Drawing ROIs directly on MRI lesions and transferring them to PET images enables precise extraction of lesional [^11^C]MeDAS uptake. Because of the unavailability of a PET/MRI camera for this study, the spinal cord was segmented per vertebra for [^11^C]MeDAS uptake extraction, which is inherently susceptible to partial-volume effects. Dynamic acquisition using an arterial input function might enhance accuracy and reproducibility of [^11^C]MeDAS PET by capturing tracer kinetics without artifacts such as flow, vascular volume, renal function, or tracer excretion rate (22–24). However, arterial cannulation for continuous sampling and the extended acquisition time increase patient burden and radiation exposure and require specialized personnel and equipment for blood-data analysis.

## CONCLUSION

[^11^C]MeDAS distribution in the spinal cord corresponds with physiologic myelin distribution and MS-related reductions in myelin density in the spinal cord. SUV derived from the ROI corresponding to 40% of the diameter of the spinal canal is the optimal parameter for quantification of myelin density in the spinal cord. Thus, [^11^C]MeDAS PET could detect significant differences in spinal cord myelin density between HCs and MS patients. The good sensitivity and NPV obtained here suggest that [^11^C]MeDAS PET can detect the absence of spinal cord abnormalities. However, poor to moderate PPV and sensitivity suggest that [^11^C]MeDAS PET is poor at detecting abnormalities found on MRI, which is likely due to partial-volume effects intrinsic to the delineation method applied.

## DISCLOSURE

This study was supported by the Nederlandse organisatie voor gezondheidsonderzoek en zorginnovatie and Stichting MS Research (grant number PTO-95105010). No other potential conflict of interest relevant to this article was reported.

## Notes

KEY POINTS **QUESTION:** Can [^11^C]MeDAS PET be used for myelin imaging of the spinal cord? **PERTINENT FINDINGS:** This prospective study showed that reduced myelin density within spinal cord lesions in animal models could be detected with [^11^C]MeDAS PET. The physiologic myelin gradient in the spinal cord in humans is detectable with [^11^C]MeDAS PET, and HCs have higher [^11^C]MeDAS uptake in the spinal cord than patients with MS, but detection of individual MS lesions in the human spinal cord is challenging. **IMPLICATIONS FOR PATIENT CARE:** [^11^C]MeDAS PET is a promising method for evaluating myelin density of the spinal cord. Such a method may be used to evaluate the efficacy of new therapies that aim to stimulate myelin repair.
